# Adverse Life Events: Do Home Care Clients Have Resources for Mastering Them?

**DOI:** 10.3389/fmed.2021.522410

**Published:** 2021-03-05

**Authors:** Vjenka Garms-Homolová, Anja Declercq, Harriet Finne-Soveri, Nanna Notthoff, Henriëtte G. van der Roest, Hein P. J. van Hout

**Affiliations:** ^1^Hochschule für Technik und Wirtschaft Berlin, University of Applied Sciences, Berlin, Germany; ^2^Lucas KU, Leuven, Belgium; ^3^Institute for Health and Welfare, Helsinki, Finland; ^4^Faculty of Sport Science, Leipzig University, Leipzig, Germany; ^5^Netherlands Institute of Mental Health and Addiction (Trimbos-Institute), Utrecht, Netherlands; ^6^Amsterdam University Medical Center, Vrije Universiteit, Amsterdam, Netherlands

**Keywords:** major life stressors, depression, stability of health status, social resources, home care clients, functional dependency, interRAI-assessment, European study

## Abstract

**Objectives:** Research on life stressors and adverse life events has a long tradition. Few studies have addressed this topic in connection to very old people. Life stressors, especially major life stressors (MLSs) experienced by clients of home care services in the community have rarely been the subject of studies. Considering this gap, we investigated the prevalence of MLSs in home care clients. We examined the effects that MLSs have on their mood and health status as well as the impact of clients' social resources on MLSs and their outcomes.

**Method:** We used assessment data from 2,884 home care clients in six European countries. The methodological basis was the comprehensive and standardized interRAI Home Care Assessment (interRAI HC).

**Results:** Fifteen point four percent of the sample—that consisted of women and men with an average age of 82.89 years–experienced an MLS in the last 6 months before the assessment. They were more depressed than persons without these experiences, and their health status indicated a higher level of instability and deterioration. At reassessment after 6 months, the situation changed. Despite the fact that both outcomes of the MLSs, depression and health status became worse in the reassessment-sample, home care clients without MLS were more affected by the worsening, especially that of depression. The expected buffering impact of social resources was low.

**Discussion:** Although this study worked with limited information on MLSs, it could contribute to closing various knowledge gaps. The study shows that the MLSs represent a prevalent problem in a population of home care clients and that this problem has negative consequences for their mood and the stability of their health status. Furthermore, this research took up the situation of very old and vulnerable adults, who have previously rarely been considered in studies on major critical life events and stressors.

**Conclusion and Research Perspective:** Future research on MLSs has to take up the issue of the time passage between the MLS and the impact on health and well-being of individuals dependent on care. It has to determine immediate as well as later consequences and identify those factors that are appropriate to reduce the MLS-effects on very old people dependent on care.

## Introduction

Research on critical life events and life stressors has generated a respectable body of knowledge. However, when studies on life stressors in health sciences and psychology are considered more closely, knowledge gaps become obvious.

Since the nineteenseventies, researchers have investigated events that cause a considerable change of life (1, 2). They have concentrated not only on negative and stressful experiences, but also on positive life changes, such as the birth of a child, job-related advancement, etc. Both types of changes have been recognized as having a pathogenic impact on the life course and overall health (3). Both have been identified as powerful risk factors for cardiovascular disease and hypertension (4). Researchers have developed typologies of people in middle age. Problems of behavior regulation, coping, and overcoming the stressors entered the research agenda (5–7). Yet, the risk of functional dependency in older people as a consequence of the life events was not considered in those days.

Later, the association between critical life events and depression moved to the foreground. Critical life events as flash points of mental illnesses (not only of depression) became an important subject of studies on clinical populations, for instance, on small samples of hospitalized psychiatric patients. Mazure (8) reported on 14 studies on life events and stressors completed between 1980 and 1990. Most of them worked with ~33 participants, the largest one with 227 participants. Larger samples were addressed by some community-based studies (9). The common targets were younger females, university students, or children. The researchers developed diagnostic instruments and treatment strategies just for these groups.

Over time, a large number of possible life stressors has been described (10–12): death of a family member or close relative or other important person, divorce or separation from partner, loss of a friend, legal problems, serious health crises, e.g., notification of a serious diagnosis, or financial problems. Major stressors in older age include bereavement, financial loss, new physical illness or disability of oneself or of a family member, change in living situation, and interpersonal conflict (12). Bellingtier et al. (13) relate MLSs to the subjective experience of age (subjective age).

Despite this tradition and the large amount of existing studies, neither a unified definition of the term major life stressor (MLS) nor a consistent concept exist. A variety of terms overlap (10). Some authors speak about “traumatic events” (14) having the potential to affect psychological well-being, about “critical life events,” or about “adverse life experiences” [(8), p. 291, 294]. Still others use “uncontrollable events” that have adverse biological and behavioral outcomes or the terms “loss” and “exit events” (15). Such variety reflects an ambiguity of concepts. Accordingly, a comparison of outcomes is difficult, and the evaluation of different stressors for individual people is hardly possible. Above all, the effects of short-term, acute stressors, which probably constitute the majority of the MLSs are less known (16). Studies with community dwellers in their normal environment are scarce. Research concentrates on the clinical environment of hospitals, on educational institutions, or on laboratory settings (17). As a consequence, little is known about the meaning of life stressors for older people in their everyday environment.

Researchers distinguish between acute and chronic stressors [(18), p. 174]. Chronic stressors are conceived as discrete events and conditions, or constellations of related events that persist over time (17). In the healthy population, both acute as well as chronic stressors seem to be associated with depression (19), with chronic stressors having a pronounced impact (20). As far as elderly people who already suffer from depression are concerned, both chronic and acute stressors seem to predict an increase in depression (11). However, in the context of long-term care, the topic of chronic stressors has almost exclusively been investigated in informal or professional caregivers of the elderly care receivers, not the elderly themselves (21–25).

Some studies have stressed a buffering or moderating effect of social support in the “stress – depression” relationship [(18), p. 177]. Independent causal effects were found in older women with depressive symptoms and in bereaved or physically disabled persons (26, 27). Tennant (18), who summarized corresponding findings underlined that buffering effects of social support and social relations were identified in children, adolescents, and young people, such as university students.

Against this background of existing research, our paper will direct the attention to issues that have not played a big role in available studies: life stressors in to date rather “invisible” clients of home care, very old and functionally dependent people who live in the community and are not institutionalized. Thus, we focus on the naturalistic context of long-term care, which is the prevailing form of care provision in European countries.

Our research questions are:

How prevalent are major life events in an international sample of home care clients?
Does the level of the home care clients' dependency reduce the frequency of MLSs recorded by the assessment?
Do the MLSs affect mood and health status of the home care clients?
Do social resources buffer against negative MLS-outcomes, immediately and over the course of 6 months?

## Materials and Methods

We used data from 2,884 home care clients who underwent a standardized geriatric assessment (interRAI HC—see www.interRAI.org) and two reassessments, the first one (T_1_) after 6 months, and the second one (T_2_) after 1 year. This paper is based on the baseline (T_0_) and the first reassessment (T_1_). The study was carried out in six European countries: Belgium (B), Finland (Fin), Germany (De), Iceland (Ice), Italy (I), and the Netherlands (NL). More details about the entire IBenC-project are described elsewhere (28). Some information on the sampling procedure should be given here. In a first step, typical home care organizations were selected in the participating countries. In a second step, receivers of home care, home nursing, and home help were recruited. Only persons who were 65 years of age and older and who already were clients of the service for at least 14 days were included (Table 1). Persons with explicitly negative prospects for the next 6 months were excluded, i.e., clients receiving palliative care or end of life care, and people facing an impending ending of home care because of the admission to a hospital or nursing facility. In this way, a possible reduction of the samples between T_0_ and T_1_ was to be prevented. In the Netherlands, the exclusion criterion “probability of nursing home admission in the next 6 months” was implemented particularly strictly. Thus, this sample contains fewer cognitively impaired clients than samples from other countries. Moreover, access to home care for less impaired persons was relatively easy at the time of the assessment in the Netherlands. The fact that the Dutch sample contains less severe cases is reflected by some results (Table 1). Therein, it is apparent that the mean score of cognitive performance in the sample of the Netherlands is below zero, which means “cognitively capable and independent in decision making.” The geriatric interRAI HC-assessment contains up to 320 variables covering health and functional status, cognition, mood, social capabilities, and behavior. Basic information on demography, living arrangement, and living environment is also included. Four sections are dedicated to the provision of health care.

**Table 1:** Study samples in participating countries with activities of daily living (ADL), instrumental activities of daily living—performance (IADLp), and cognitive performance (CPS) of the home care clients.

**Country**	**Number of Participants at baseline**	**% of the sample**	**ADLh: Mean (SD)**	**IADLp: Mean (SD)**	**CPS Mean (SD)**
Belgium (B)	525	18.2	3.16 (1.18)	33.99 (10.72)	1.35 (1.63)
Finland (Fin)	456	15.8	0.77 (1.31)	26.42 (13.05)	1.33(1.18)
Germany (De)	493	17.1	2.16 (1.72)	28.68 (14.85)	1.61 (1.72)
Iceland (Ice)	420	14.6	0.58 (1.06)	23.78 (11.47)	1.07 (1.17)
Italy (I)	499	17.3	3.82 (1.67)	39.34 (1.67)	2.32 (2.07)
The Netherlands (NL)	491	17.3	0.46 (1.13)	16.95 (12.26)	0.59 (.86)
Total	2,884	100	1.87 (1.90)	28.95 (14.24)	1.38 (1.60)

Differences between country-samples:

*ADL: square sum = 4,950.571, df = 5, square middle = 990.114, F = 7,523.271, p < 001*.

*IADL: square sum = 128,308.645, df = 5, square middle=25,660, F = 167,281, p < 001*.

*CPS: square sum = 805.448, df = 5, square middle = 161.090, F = 70.871, p < 001*.

The assessments were carried out by professional nurses who provided the normal, everyday care. In some countries, “routine data” were used. This term means that the data were collected in the course of regular health care and nursing. To ensure good quality of data, a special training was offered to this staff. Additionally, research nurses were available to support the nurses during the process of data collection. The procedure of the interRAI HC assessment is special. The assessors link three to four sources of information: their professional evaluation of the status and situation of the individual, self-reports of the persons under study, information documented in clients' records, and sometimes also the evaluation by other persons involved with the case of the client.

For the purposes of this paper, only selected variables and scales were analyzed. The scales are constructed with interRAI HC-variables.

Major life stressor in the last 90 days is a dichotomous variable. Participants reported whether or not they had experienced a major life stressor in the last 90 days. The assessment manual describes the MLS as an “episode of severe personal illness, death or severe illness of a close family member or friend, loss of the person's home, major loss of income or assets, being a victim of a crime, e.g., robbery or assault.” Usually, such experiences disrupt or threaten to disrupt a person's daily routine and impose a readjustment (29).

The following measures were considered dependent variables, or outcomes in the present paper.

*Depression Rating Scale:* DRS describes the mood status of the clients (30). It ranges from “0” (no indication of mood problems or depression) to “14.” A score of “3” indicates minor, and higher scores indicate major depressive disorder.

The *CHESS scale* (full name Changes in Health, End-Stage Disease, Signs, and Symptoms) is a six-point scale that helps to identify individuals whose health status is highly unstable and who are at risk of serious decline. It ranges from “0” (not at all unstable) to “5” (highly unstable), and the highest levels predict mortality, hospitalization, negative subjective health ratings, and other health deterioration (31).

The demographic characteristics considered in the present manuscript were age (in years), gender (male or female), marital status (never married, married or partnership, divorced, separated), and living arrangement (number of persons living with the client).

Clients' dependency was measured by ADL and IADL indices as well as by frequency and amount of home care and help services.

*IADL “Performanc*e” reflects the level of actual execution of the instrumental tasks of daily life by the client. The score ranges from “0” to “48” (32).

*Functional performance* in elementary activities of daily life is measured by the ADL hierarchy scale (ADLh) with seven levels, starting with “0” (independent) and ending with “6” (totally dependent) (32).

*Services*: We took the frequency and amount of services that help with completion of the everyday tasks as further indicators of dependency. We selected variables indicating the frequency of home health care and homemaking. Our question was: “On how many days of a week do clients receive these services?” Additionally, we used variables indicating the amount of both services (in numbers of minutes of the provision per week).

Social resources were measured by six variables:

*Mutual visits* with a family member and long-standing friends. Possible responses ranged from never (0) to more than 30 days ago (1) to 30 days ago (2), etc., up to in the last 3 days (4).

*Social interaction* with family member and long-standing friends by telephone, e-mail, etc. The characteristics and response categories were the same as for the variable “visits.”

*Availability of a strong relationship* with the family, which makes it possible that the person “feels able to rely on family members” (29).

*Living arrangement* (contains information about living alone or with one or more other people).

*Number of informal helpers*.

*Believes in improvement potential*: This variable contains two perspectives: the client's own belief that he/she will improve his/her status and physical performance, on the one hand, and the beliefs of the professional caregivers in the improvement potential of the client, on the other hand.

### Potentially Stressful Factors

We used the *Pain scale* that measures the intensity and frequency of pain during the last three days before the assessment. It ranges from “0” (no pain) to “3” (daily severe pain) (33).

*Problems in social relations* were measured by three continuous variables: Conflict or anger with family or friends; fear of a family member or close acquaintance; neglect, abuse, or mistreatment. Possible responses were never (0), more than 30 days ago (1), 30 days ago (2), etc., up to “in last 3 days” (4).

**Cognitive status** was measured by the *Cognitive Performance Scale* (CPS), with seven levels and especially based on daily decision making, but including short-term memory and other items as well (34). The first level is “0” (cognitive performance is intact), the highest level is “6” (very severe impairment of cognitive performance).

## Results

The entire study sample contained 2,884 home care clients, 67.4% of which were women. The proportion of participants from the six countries ranged from 14.6% in Iceland to 18.2% in Belgium (see Table 1). The average age of the home care clients under study was *M* = 82.89 years (*SD* = 7.26; *Md* = 84.00, *age range* 65–105 years*)*. Germany participated with the oldest clients (*M* = 84.19 years, *SD* = 7.57). The Italian sample consisted of the youngest individuals: their average age was *M* = 81.85 years (*SD* = 7.91). Age differences were considerable and significant [*F*_(5,1)_ = 7.03; *p* < 0.001].

Despite the fact that the Italian participants were younger than participants from the other countries, they had the highest level of impairments (Table 1). The average of the ADLh-score in Italy was almost 4 (*M* = 3.82, *SD* = 1.67), which means extensive need for help. The average ADLh-score of the entire sample of all countries was only *M* = 1.87 (*SD* = 1.90), which indicates “limited need for help.” The Italian sample showed similar levels of impairment of the performance in instrumental activities and in cognition. In contrast, the Dutch home care clients were almost independent in ADLh (*M* = 0.46, *SD* = 1.13) and an average CPS-score that indicates that the cognitive performance of the Dutch home care clients was practically unimpaired (*M* = 0.59, *SD* = 0.86).

### Prevalence of Major Life Stressors Within 90 Days Before the Assessment

At baseline, 15.40% of the clients (445 persons) reported that they experienced an MLS in the past 90 days before the assessment (Table 2). The frequencies differed significantly from country to country [χ^2^(5) = 125.02, *p* < 0.001]. 31.20% of the Dutch home care clients experienced an MLS. This figure is far above average. In contrast, the proportion of Finnish home care clients who experienced a major stressor was below average with 7.50%.

**Table 2:** Differences of the potential outcome scales (DRS and CHESS) in country-samples.

**Country**	**% of clients with MLS in the last 90 days prior the assessment**	**DRS: mean- score (SD)**	**Chess: mean-score (SD)**
Belgium (B)	16.5	1.76 (2.53)	1.09 (1.02)
Finland (Fin)	7.5	0.93(1.86)	0.68 (.90)
Germany (De)	13.2	1.59 (2.61)	0.65 (.94)
Iceland (Ice)	11.7	1.16 (1.82)	1.15 (.97)
Italy (I)	12.2	1.32 (2.03)	1.64 (1.27)
The Netherlands (NL)	31.2	1.60 (2.15)	1.23 (.99)
Total	15.6	1.41 (2.22)	1.07 (1.08)

Differences between country-samples:

*DRS: square sum = 233.232, df = 5, square middle = 46.646, F = 9.589, p = 000*.

*CHESS: square sum = 327.650, df = 5, square middle = 65.530, F = 61.969, p = 000*.

The likelihood of experiencing an MLS was higher in the Netherlands than in Germany (reference country) [*OR* = 2.97, *S.E. (B)* = 0.17, *Wald* χ^2^ = 43.63, *p* < 0.001]. The likelihood of reporting an MLS was lower in Finland than in Germany [*OR* = 0.53, *S.E. (B)* = 0.22, *Wald* χ^2^ = 8.18, *p* = 0.004].

### Association of MLSs and Demographic Characteristics

Home care clients who experienced a major life event in the last 90 days were slightly and significantly younger: Their average age amounted to *M* = 82.18 years (*SD* = 7.04), whereas persons who did not report an MLS were *M* = 83.02 years old (*SD* = 7.30), on average. [*F*_(1,2842)_ = 5.10; *p* < 0.05]. This association was not found in all participating samples. In those samples, where the proportion of MLS reports was especially high (Dutch sample) or especially low (Finland), no significant association between MLS and age was found.

The prevalence of MLSs did not differ between men and women. Likewise, there was no significant difference by living arrangement (number of persons living in the household of the client). However, among the divorced and separated clients, the number of persons who reported an MLS was higher than in those who were married and single [χ^2^*(3)* = 10.69; *p* < 0.05].

### Factors, Which Potentially Could Be Experienced as Stressful

We focused on pain and on problems of social relations. The average Pain-score of home care clients without the experience of an MLS was *M* = 0.79 (*SD* = 0.97); the average Pain-score of clients with MLS was higher (*M* = 1.08, *SD* = 1.14, t_(2836)_ = −5.57; *p* < 0.001).

We ran a logistic regression with MLS as the outcome and pain as the predictor. The higher the Pain-score, the greater the likelihood was that participants reported an MLS [*OR* = 1.30, *S.E.(B)* = 0.05, *Wald* χ^2^ = 30.15, *p* < 0.001]. There was no interaction between Pain-score and the belonging to a country-specific subsample, i.e., the effect of pain on likelihood of reporting an MLS did not differ by country.

*Problems of social relations* which potentially could be stressful: We focused on three variables. As far as persisting conflicts with family or friends were concerned, their presence was relatively more frequent in clients with MLS than without [χ^2^(2) = 27.34; *p* < 0.001]. However, it was irrelevant when such conflicts took place: The temporal distance between the conflicts and the assessment was not related to the occurrence of the MLSs.

### Physical Dependency Does Not Reduce the Probability of Assessed MLSs, Cognitive Impairment Does

We ran a logistic regression with MLS as outcome and with ADLh scale and IADL performance scale as predictors. ADLh-score and IADL-score were unrelated to the probability of reporting an MLS.

A different picture emerged when impairment of cognitive performance was considered. The logistic regression with MLS as outcome and CPS as predictor showed that the likelihood of reporting an MLS decreased with increasing CPS-score (i.e., with the increase of cognitive impairment) [*OR* = 0.87, *S.E.(b)* = 0.04, *Wald*(1) = 13.90, *p* < 0.001]. We did not find an interaction between CPS and country, i.e., the effect of CPS on reporting of MLS did not differ between the country subsamples.

A logistic regression with MLS as outcome and frequency and amount of services as predictors showed: The higher the number of days of home health care in the past 7 days was, the greater was the probability that MLS would be recorded in the assessment [*OR* = 1.05, *S.E.(b)* = 0.02, *Wald(1)* = 4.79, *p* < 0.05]. The higher the number of minutes of homemaking services in the past week was, the higher was the probability that MLS would be captured by the assessment [*OR* = 1.00, *S.E.(b)* = 0.08, *Wald* (1) = 20.89, *p* < 0.001].

### Outcomes of Experiencing the MLS

*Depression as outcome*: A clear difference was identified in mood status. Home care clients who experienced MLS were more depressed than those who did not. We conducted an independent samples *t*-test. Participants with MLS had a higher DRS-score (*M* = 2.24, *SD* = 2.62) than participants without MLS (*M* = 1.25, *SD* = 2.10), *t*_(2843)_ = −8.73, *p* < 0.001.

*Stability of health status as outcome:* The CHESS-score as an indicator for the instability and decline of health status showed differences between home care clients with and without MLS. We conducted an independent samples *t*-test. Clients with MLS had a higher CHESS-score (*M* = 1.44, S*D* = 1.16) than clients without MLS (*M* = 1.01, *SD* = 1.05), *t*_(2827)_ = −7.81, *p* < 0.001.

### Influence of Social Resources on MLS-Outcomes

The majority of the home care clients maintain social relations with family members, friends, and other significant persons. Only 4.60% never cultivated mutual visits with these people; only 6.40% never communicated on the phone, via email, etc. 48.80% had such mutual visits in the last 3 days before the assessment, 16.70% in the last week, 10.00% in the last 2 weeks, and 4.60% a longer time ago. The communication by phone or digital tools occurred with about the same frequency: 47.00% communicated during the last 3 days, 13.00% in the last week, 7.10% in the last 2 weeks, and the rest a longer time before they underwent the interRAI HC-assessment. Home care clients with MLS reported more recent visits and interactions. Fifty six point six percent participated in a visit in the last 3 days (*K* = 0.60, *p* < 0.05), and 58.70% communicated with a close person in the last 3 days (*K* = 0.09, *p* = 0.001).

Neither visits [*b* = −0.01 *SE(b)* = 0.09, *F*_(1,2625)_ = 0.86, n.s.] nor other interactions with family and close friends [*b* = −0.02 *SE(b)* = 0.08, *F*_(1,2625)_ = 0.27, n.s.] had a buffering effect on depression (DRS-score).

Only two single social variables seemed to have some buffering impact on depression. On the one hand, it was the number of informal helpers. The more informal helpers participants had, the less depressed they reported being after a major life event [*b* = 0.33, *SE(b)* = 0.16, *F*_(1,2636)_ = 4.38, *p* < 0.05]. On the other hand, the self-evaluation of the improvement potential played a role: DRS-score was higher, if clients did not believe that their physical function could improve, whereas in participants with MLS, DRS was higher, if they thought that their physical function could improve [*b* = 0.88, *S.E.(b)* = 0.34, *F*_(1,2625)_ = 6.47, *p* < 0.05]. No meaningful influence of social resources on health status (stability measured by the CHESS-score) in terms of buffering was identified. Visits [*b* = 0.01 *SE(b)* = 0.04, *F*_(1,2584)_ = 4.45, *p* < 0.05] had little effect on “health status instability” (CHESS-score) since the effect size was very small (partial η^2^ < 0.01), and the confidence interval of the parameter estimates included 0 (−0.07, 0.01). The item “other interactions with family and close friends” seemed to have a detrimental effect [*b* = −0.05 *SE(b)* = 0.04, *F*_(1,2584)_ = 9.15, *p* < 0.05], but again, the effect size was very small (partial η^2^ <0.01), and the confidence interval included 0 (−0.12, 0.03).

### Changes Over the Course of 6 Months

We examined changes over the course of 6 months, i.e., from baseline (T_0_) to the first reassessment (T_1_) in persons who survived and were not discharged during these 6 months. Were some of the social resources from baseline still important for buffering against depression (as indicated by the DRS-score) and the instability of health status (CHESS-score) at the first reassessment?

First, we examined changes of the DRS-score from baseline to the first reassessment. It clearly increased from T_0_ to T_1_ [*M*_*d*_ = 0.30, *SD* = 2.05, *t*_(1971)_ = 6.54, *p* < 0.001]. However, the DRS-score increased more in participants without MLS (*M*_*d*_ = 0.35, *SD* = 1.95) than in participants with MLS (*M*_*d*_ = 0.03, *SD* = 2.53) at T_0_; this difference was statistically significant, *t*_(1964)_ = 2.47, *p* = 0.014.

Afterwards, we tested, what effects social resources exhibited with regards to changes in DRS-score of people who experienced MLSs and those who did not. Our attention was directed to the following variables: strong relationship with the family, living together with one or more other people, mutual visits with family/friends, interaction by phone or email, number of helpers, own and staff's assessment of a potential for improvement. These resources did not play a role.

In a second step, we controlled for changes of the CHESS-score from baseline to the first reassessment. It changed similarly to the DRS-score. Health status became more unstable over the course of 6 months between the assessments T_0_ and T_1_, as the CHESS-score went up [*M*_*d*_ = 0.15, *SD* = 0.88, *t*_(1950)_ = 7.38, *p* < 0.001]. In participants without major life event, the CHESS-score increased (*M*_*d*_ = 0.18, *SD* = 0.87), whereas in participants with MLS at T_0_, the CHESS-score decreased, or more accurately, it almost stayed the same (*M*_*d*_ = −0.05, *SD* = 0.90). Changes in CHESS-score differed significantly between participants with and without MLSs [*t*_(1942)_ = 4.03, *p* < 0.001].

We tested what role social resources played for effects of MLS on changes in the CHESS-score. Across the whole sample, there was no effect of social resources on changes in CHESS-score from T_0_ to T_1_. For people who reported MLS at baseline, the availability of social resources played a role. The more social resources in form of mutual visits with family or other social contacts these clients had, the more their CHESS-score increased from T_0_ to T_1_. It means that the health status became more unstable just in those home care clients with MLS at T_0_, who had social resources at their disposal. In clients without MLS at baseline, social resources were unrelated to changes in CHESS-score [*b* = −0.11, *S.E.(b)* = 0.05, *F*_(1,1745)_ = 4.28, *p* = 0.02].

We investigated, if this could have been the result of the worsening of cognitive performance, which—as we could show in section 3.3—was related to the assessment of a smaller portion of MLSs. This seemed to be the case. The general linear model of the reassessment data showed a predictive role of the CPS-score on the CHESS-score both without the MLS at baseline [*b* = 0.12; *SE(b*) = 0.02; *t* = 9.30; *p* < 0.001] and with the MLS at baseline [*b* = 0.12; *SE(b)* = 0.04; *t* = 2.96; *p* < 0.005]. The same association was not found with regards to the DRS-score, which decidedly was not dependent on development of cognitive performance over the course of 6 months since the baseline assessment.

## Discussion

This contribution that is based on a European collaborative study shed light on very old people who receive long-term care while living in the community, not in a long-term care institution. We investigated to what extend they were burdened by MLSs and which consequences it had for them. According to our assessment, 15.40% of study participants experienced an MLS within 90 days prior to the assessment. The differences between country-samples were substantial. In particular, the Dutch home care clients were very different from the clients in other countries. The MLS was assessed in almost one third of them. This finding may be caused by the slightly different sampling by the researchers in the Netherlands who—as we already explained–did not include clients with a higher level of cognitive impairment into their sample for several reasons (see Material and Methods Sections and Results, this article). But this assumption must be handled with caution. We indeed saw that the likelihood of reporting an MLS decreased with the increase of cognitive impairment; yet, we did not find any effects of CPS-score on reporting of MLSs in individual countries.

We asked, if community nurses who collected the data for the IBenC-study paid sufficient attention to the MLSs even of very dependent clients. We assumed that a high level of dependency could be distracting of such adverse experiences. Two considerations lead us to this assumption. On the one hand, not much research on how well-community nurses assess life stressors has been published until today. Préville et al. (35) underlined that psychological distress and signs of depression are not easily detected by home care nurses, since these problems often are masked among older adults, particularly among frail elderly. On the other hand, studies on life stressors in the context of long-term care rather rarely focus on care-receivers. Much more often, the caregivers are the focus. Providing care for an ill or disabled relative represents a burden for the informal caregivers. For instance, spouses or adult children are frequently exposed to stressors that may place many caregivers at risk for depression (12). Research on these problems of caregivers seems to be more important for practical reasons of developing support strategies for them. In this context, the home care receivers seem to slip the attention of researchers.

However, we could not confirm our primary assumption that a higher level of dependency could reduce the proportion of MLS-reports. The functional dependency of the clients was unrelated to the MLS-reports. With a higher frequency and greater amount of professional care and help, it was more likely that MLSs were reported and recorded in the assessment. More service seemed to be an indicator for higher level of dependency. Yet, it may be that clients have a better opportunity to verbalize critical events and stressors vis-á-vis the care staff, if they get more services. Equally, they may be able to report their MLSs also to assessors.

Only cognitive dependency showed different effects. Two possibilities should be considered: Impaired cognition may be straining and may therefore redirect staff's attention away from problems like adverse life experiences. Or, because of their reduced cognitive capacity, clients with higher levels of cognitive impairment are not capable of expressing their critical experiences and MLSs.

We saw almost no significant association between demographics and the prevalence of MLSs. Age differences were small, and gender differences were non-existent, even if some authors argued that older women are especially vulnerable to life events and stressors (36).

Our assumption that MLSs will have negative consequences was confirmed fully. The DRS-score of home care clients who experienced MLSs was higher than the DRS-score of clients without such experiences. Our data could not answer the question about causality. However, this question remains open in many studies because the onset of depression is dependent on a multiplicity of factors, including genetic factors (37). Large numbers of life stressors and critical life events are listed (12). Nevertheless, it remains difficult do decide, which of them definitely causes depression (38), and which of them is the “major” life stressor (38). The explanation varies between individuals, life contexts, and different stages of life and–according to a review of studies (18)—it varies also between types of depressive disorders. We considered the pain-status of the home care clients to be what is called the “precipitating” or “incubating” factor (39) that precedes the life stressor experiences. Indeed, we identified the pain-score as a predictor. The higher the Pain-score was, the greater was the likelihood that participants would report an MLS. Our results could confirm our assumption that persistent interpersonal conflicts with family members and other significant acquaintances may be considered chronical life stressors. They seemingly promote the appearance of the MLSs in our sample and may have the function of social precursors [(16), p. 204].

We were interested in the issues of social resources that could moderate the negative consequences of the MLSs. However, activities of maintaining social contact and interaction did not have a significant impact on outcomes “depression” (DRS-score) and “health status instability” (CHESS-score). Both scores became worse during the time between the baseline assessment and the first reassessment. This development was significantly associated with the occurrence of MLSs in the last 90 days before the baseline assessment. But the direction of the association was surprising. The DRS-score and also the CHESS-score increased in home care clients who had not experienced an MLS, whereas they were almost unchanged in clients with MLS-experience at baseline. This may be the phenomenon described by Tennant (18), who underlined that an effect of stressors often “dissipates with the passage of time” [(18), p. 175]. This phenomenon was reported in connection to the “acute stressors,” which may be similar to stressors which we call major life stressors in our investigation.

### Strengths and Limitations

Our article reports on a study that has various strengths and weaknesses. The sample was not composed to be representative for the countries. Yet, it reflects typical home care services, the typical composition of their body of clients, and finally, typical individual clients who are taken care of by the community services in six European countries for a relatively long time. The sample was recruited in a naturalistic environment of communities—a situation which is rare as some life events researchers have stated (38). Moreover, the number of study participants is larger than in many other studies (8), especially in studies dealing with stressors of older adults [e.g., (40)]. We believe that the large sample size improves the information value of our analysis.

The target group of our study are older home care clients, i.e., older than 80 years, on average. Such a population was rarely targeted by studies on life stressors and critical life events. Even studies that proclaim “aging” and “older adults” in their title, mainly investigated much younger people, for example those around 55 years of age or people who were about 77 years old (40, 41). Kraaij et al. (11) undertook a meta-analysis of studies on “negative life events in elderly persons”. It included 25 studies, but only three of them dealt with participants who were just reaching 80 years of age. Few studies focused on receivers of long-term care in the community, as our study did. Richardson et al. (40) investigated applicants for services provided by the Aging Services Provider Network. Here, clients of non-medical services, like advocacy, meals delivery, transportation, and care management, were targeted. Receivers of care or nursing were explicitly not included.

Existing studies in the long-term care environment either targeted the institutionalized population of nursing homes (42, 43) or the professional and informal caregivers of older people (22, 23, 25).

One weakness of our study is that our information on the MLSs is limited, since we only used a dichotomous variable indicating whether or not the client experienced a major life event in the last 90 days before the assessment. This weakness should be considered in view of the large diversity of the concepts of life stressors and life events. In an assessment like ours, no collection of information on details and different characteristics of life stressors was possible.

## Conclusion

Our analysis offers a first approach to the impact of MLS in this special population and is therefore an appropriate point of departure for research on this subject. Continuing research into MLSs should take up the issue of the time passage between the MLS and the impact on health and well-being of individuals dependent on care. It should attempt to determine immediate as well as later consequences and those factors that are appropriate to reduce the deterioration of very old people dependent on care.

## Data Availability Statement

The datasets generated for this study are available on request to the corresponding author.

## Ethics Statement

The studies involving human participants were reviewed and approved by the ethics committees of the institutions of all contributing study directors. The patients/participants provided their written informed consent to participate in this study.

## Author Contributions

HH, AD, VG-H, HF-S, and HR: design of the study, data collection. VG-H: paper design. VG-H and NN: data analysis, first draft of the manuscript. All authors contributed to the article and approved the submitted version.

## Conflict of Interest

The authors declare that the research was conducted in the absence of any commercial or financial relationships that could be construed as a potential conflict of interest.

## Abbreviations

ADLh: Activity of Daily Living hierarchy scale
B: Belgium
CHESS: Changes in Health, End-Stage Disease, Signs, and Symptoms
CPS: Cognitive Performance Scale
De: Germany
DRS: Depression Rating Scale
Fin: Finland
HC: Home Care
I: Italy
IADL: Instrumental Activity of Daily Living Scale
Ice: Iceland
MLS: Major Life Stressor
NL: The Netherlands
T_0_: Baseline
T_1_: First reassessment
T_2_: Second reassessment.
